# Effects of dietary supplementation with microencapsulated *Galla chinensis* tannins on growth performance, antioxidant capacity, and lipid metabolism of young broiler chickens

**DOI:** 10.3389/fvets.2023.1259142

**Published:** 2023-10-26

**Authors:** Xiaojie Ren, Peng Yuan, Jiaxing Niu, Yang Liu, Yang Li, Libo Huang, Shuzhen Jiang, Ning Jiao, Xuejun Yuan, Junxun Li, Weiren Yang

**Affiliations:** ^1^Key Laboratory of Efficient Utilization of Non-grain Feed Resources (Co-Construction by Ministry and Province), Ministry of Agriculture and Rural Affairs, Department of Animal Science and Veterinary Medicine, Shandong Agricultural University, Tai’an, China; ^2^Shandong Taishan Shengliyuan Group Co., Ltd, Tai’an, China; ^3^Division of Animal and Human Health Engineering, Department of Biosystems, KU Leuven, Heverlee, Belgium; ^4^College of Life Sciences, Shandong Agricultural University, Tai’an, China

**Keywords:** antioxidant capacity, broiler, Galla Chinensis, lipid metabolism, tannic acid

## Abstract

This study aimed to investigate the impacts of dietary supplementation with *Galla chinensis* tannins (GCT) on the growth performance, antioxidant capacity, and lipid metabolism of young broilers. Overall, a total of 216 healthy 1 day-old broilers were randomly allocated to CON group and GCT group, and provided with a basal diet or a basal diet added with 300 mg/kg microencapsulated GCT, respectively, in a 21 days trial. Our findings indicated that dietary GCT addition had no significant effects (*p* > 0.05) on growth performance. However, GCT supplementation led to a significant reduction in the total cholesterol (TC) concentration in the serum and liver (*p* < 0.05). Furthermore, GCT supplementation significantly increased the ratios of high-density lipoprotein (HDL) to low-density lipoprotein (LDL) and HDL to TC in the serum, in addition to elevating the activities of enzymes related to lipid metabolism in the liver (*p* < 0.05). Dietary GCT addition also improved the antioxidant capacity of the broilers, as evidenced by a significant decrease in the concentration of malondialdehyde in serum and liver (*p* < 0.05). Additionally, the GCT group exhibited significantly increased expressions of hepatic genes associated with antioxidant enzymes (*HO-1*, *GPX1*, *SOD2*, *SIRT1*, *CPT-1*, and *PPARα*) (*p* < 0.05), while the mRNA expression of *SREBP-1* was significantly decreased (*p* < 0.05) compared with the CON group. In conclusion, dietary addition of 300 mg/kg microencapsulated GCT improved the antioxidant status and lipid metabolism of broilers without affecting their growth performance.

## Introduction

With the ongoing intensification of the poultry industry in the modern world, broilers are confronted with a multitude of challenges including infections, oxidative stress, and lipid accumulation (1, 2). These factors can disrupt the balance between oxidation and the antioxidant defense system, thereby affecting the growth performance of broilers (3, 4). Furthermore, the body’s antioxidant efficiency is closely associated with lipid metabolism. The liver, as the main metabolic organ, plays a crucial role in both the antioxidant defense mechanism and lipid metabolism. Fatty acids obtained from the gastrointestinal tract are transported directly to the liver, where over 90% of the broilers’ body fat is synthesized (5, 6). Excessive hepatic lipid accumulation can lead to severe metabolic disorders, tissue damage, and even mortality (7). Young broiler chickens (1–21 days of age) particularly susceptible to oxidative stress due to the incomplete development of their antioxidant system (8). Therefore, it is crucial to regulate antioxidant efficiency and lipid metabolism to maintain good health and prevent diseases in young broiler chickens (9, 10).

Tannins are plant secondary metabolites that can be classified into hydrolyzable tannins and condensed tannins, and they are present in various plant components including seeds, flowers, leaves, roots, and fruits (11, 12). Galla chinensis (GC), renowned in traditional Chinese medicine for centuries, contains hydrolyzable tannins as its primary bioactive compound (13). According to reports, tannins possess numerous biological properties, including antibacterial, anti-parasitic, antioxidative, anti-inflammatory, and antiviral actions (14). Tannins are highly effective and safe, causing minimal side effects and toxicity. As a result, they are frequently employed in the prevention and treatment of lipid metabolism disorders (15). Nevertheless, tannins may reduce feed intake by reducing the palatability (16). Microencapsulation is a widely used technique in the feed industry that effectively alters the absorption site of additives and conceals undesirable tastes and odors. A previous study in weaning piglets demonstrated that supplementing microencapsulated GC tannins (GCT) at doses of 500–1,000 mg/kg had beneficial effects on intestinal development and function as well as the proliferation of beneficial bacteria proliferation (17). Furthermore, supplementation of microencapsulated GCT at a dose of 300 mg/kg demonstrated several beneficial effects in broilers, representing as improving growth performance, enhancing liver function, and providing protection against lipopolysaccharide-induced liver damage by inhibiting the TLR4/NF-κB pathway (18, 19). However, there is limited scientific literature available on the effects of dietary GCT addition on the growth performance, antioxidant capacity, and lipid metabolism of young broiler chickens.

Therefore, this study aimed to investigate the impacts of dietary addition with GCT on growth performance, antioxidant capacity, and lipid metabolism of young broilers, supplying important knowledge on the role and mechanisms of microencapsulated GCT in regulating antioxidant capacity and lipid metabolism in poultry production.

## Materials and methods

### Animals and treatments

A total of 216 healthy Arbor Acres (AA) chickens (1 day of age) with an initial body weight (BW) of 48.94 ± 0.34 g were randomly assigned to two dietary groups (6 replicates per group and 18 birds each replicate) in a 21 d trial. The experimental diets included a basal diet (CON group) and a basal diet added with GCT at a dose of 300 mg/kg (GCT group), according to the procedure described in previous studies (18, 19). The GCT used in this study, with an effective tannin concentration of 40%, was supplied by the Wufeng Chicheng Biotechnology Co., Ltd. (Yichang, China). In order to improve diet palatability and reduce potential irritation caused by tannins, microencapsulation was employed to mitigate undesirable effects (17). The basal diet (Table 1) was formulated in accordance with the nutrient requirements established by the National Research Council (NRC, 1994) (20). All the broilers were kept in a mental chicken coop (three-tiered cages) positioned in an environment-controlled room and under constant lighting. The temperature in the room was kept at 32°C for the first 3 days before dropping by 1°C every other day thereafter. The weight of the broiler chickens was measured at the beginning and end of the experiment, and daily feed intake were recorded during the experiment. Average daily gain, average daily feed intake (ADFI), and feed conversion ratio (FCR) were calculated as described in previous study (18).

**Table 1:** Ingredients composition and nutrient levels of basal diets (as-fed basis).

Items	Content
Ingredients, %	
Corn	55.91
Soybean meal, 44% CP	13.78
Wheat bran	11.98
Corn starch residue	7.99
Corn gluten meal	3.99
Extruded soybean	1.50
Limestone	1.70
Calcium monophosphate	1.10
L-Lysine HCl, 76.8%	1.00
DL-Methionine, 98%	0.20
L-Threonine, 98%	0.10
Sodium chloride	0.40
Choline	0.10
Phytase	0.10
Complex enzyme	0.02
Trace mineral premix^a^	0.10
Vitamin premix^b^	0.02
Antioxidant	0.02
Total	100
Calculated analysis, %	
Metabolizable energy, MJ/kg	12.33
Crude protein	19.47
Crude fat	3.45
Calcium	0.94
Available phosphorus	0.35
Lysine	1.15
Methionine	0.50

a Provided per kilogram of complete basal diet: 10 mg of Cu as CuSO_4_, 100 mg of Fe as FeSO_4_, 1.1 mg of I as Ca(IO_3_)_2_, 65 mg of Zn as ZnSO_4_, 100 mg of Mn as MnSO_4_ and 0.3 mg of Se as Na_2_SeO_3_.

b Provided per kilogram of complete basal diet: vitamin A 10,000 IU, vitamin D_3_ 3,000 IU, vitamin E 30 IU, menadione 1.3 mg, thiamine 2.2 mg, riboflavin 8 mg, pyridoxine 4 mg, vitamin B_12_ 0.025 mg, D-biotin 0.2 mg, niacin 40 mg, folic acid 1 mg, and D-calcium pantothenate 10 mg.

### Samples collection

On day 21, one broiler in each replicate (cage) with a BW close to the average of the replicate were selected after a 12 h fasting period. Blood samples were collected from the wing vein into the vacuum tubes, and the serum was then obtained by centrifugation (3,500 × g, 15 min), followed by being stored at −20°C for further analysis. Subsequently, the broilers were euthanized by cervical dislocation and eviscerated. Approximately 5 g of liver samples were collected and stored at −80°C after quick-freezing in liquid nitrogen; and another part was fixed with 4% paraformaldehyde solution for 24 h at room temperature after being rinsed with normal saline.

### Determination of serum biochemical parameters

The serum concentrations of total protein (TC), triglyceride (TG), low-density lipoprotein (LDL), and high-density lipoprotein (HDL) were examined using an automatic biochemical analyzer (COBUS MIRA Plus, Roche Diagnostic System Inc., United States) with commercially available kits (Jiancheng Bioengineering Institute, Nanjing, China).

### Determination of antioxidant capacity

Samples of liver tissues were homogenized with ice-cold 0.9% sodium chloride (1,10, w/v), and centrifuged at 4,000 rpm at 4°C for 10 min to obtain clarified homogenates. Activities of glutathione peroxidase (GSH-Px), total superoxide dismutase (T-SOD), and catalase (CAT), as well as the levels of total antioxidative capacity (T-AOC) and malondialdehyde (MDA), in serum and liver samples were determined using kits purchased from Jiancheng Bioengineering Institute in accordance with the methods described by Chen et al. (21). The concentration of liver hydrogen peroxide (H_2_O_2_) was assayed using commercial kits (Beyotime Biotechnology, Shanghai, China) following the manufacturer’s protocol (22).

### Liver morphological analysis

After 24 h fixation with 4% paraformaldehyde solution, the liver tissues were dehydrated with graded concentrations of ethyl alcohol, and embedded in liquid paraffin (23). Then 5 μm slices of the liver tissue were cut and stained with hematoxylin and eosin (H&E) after being embedded in paraffin wax. And liver sections were observed under an Olympus digital microscope (Olympus BX51, Tokyo, Japan).

### Determination of liver lipid metabolism-related parameters

The levels of triglyceride (TG), total cholesterol (TC), total lipase (TL), lipoprotein lipase (LPL), and hepatic lipase (HL) in liver homogenate samples were assayed using the commercial kits purchased from the Jiancheng Bioengineering Institute. The activities of fatty acid synthase (FAS), acetyl CoA carboxylase (ACC), and hormone-sensitive TG lipase (HSL) in hepatic were determined using kits from Yili Biological Technology Co., Ltd. (Shanghai, China).

### Gene expression

The TRIzol Reagent (Invitrogen, Carlsbad, CA, United States) was used to extract the total RNAs from the frozen liver samples according to the manufacturer’s instructions. A reverse transcription kit (TaKaRa, Dalian, China) was used to synthesize cDNA, which was then amplified by quantitative real-time PCR using SYBR Premix Ex Taq Reagents (TaKaRa). The primer sequences were displayed in Table 2, and β-actin was quantified in parallel as the internal control for normalization and quantification of transcription levels. The PCR cycling conditions were set as described in a previous study (3). The 2^−ΔΔCt^ method was applied to determine the relative abundances of the mRNA of the detected genes in liver samples.

**Table 2:** Primer sequences used for quantitative real-time PCR.

Genes^b^	GenBank	Primer sequences^a^, 5′-3′^1^	Size, bp
*β-actin*	NM_205518.1	F:ATTGTCCACCGCAAATGCTTC R:AAATAAAGCCATGCCAATCTCGTC	113
*NRF2*	XM_015289381.2	F:CCCGCACCATGGAGATCGAG	180
		R:GGAGCTGCTCTTGTCTTTCCT	
*HO-1*	NM_205344.1	F:GTCGTTGGCAAGAAGCATCC	106
		R: GGGCCTTTTGGGCGATTTTC	
*SOD1*	NM_205064.2	F:CGCAGGTGCTCACTTCAATCC R:CAGTCACATTGCCGAGGTCAC	89
*SOD2*	NM_204211.2	F:GCTGTATCAGTTGGTGTTCAAGGA R:GCAATGGAATGAGACCTGTTGTTC	130
*CAT*	NM_001031215.2	F:GGAGGTAGAACAGATGGCGTATG R:CGATGTCTATGCGTGTCAGGAT	114
*GPX1*	NM_001277853.3	F:CGGCTTCAAACCCAACTTCAC R:CTCTCTCAGGAAGGCGAACAG	85
*SIRT1*	XM_046920057.1	F:GATCAGCAAAAGGCTGGATGGT	143
		R:ACGAGCCGCTTTCGCTACTAC	
*CPT-1*	NM_001012898.1	F:ACAGCGAATGAAAGCAGGGT	93
		R:GCCATGGCTAAGGTTTTCGT	
*PPARα*	NM_001001464.1	F:AGTAAGCTCTCAGAAACTTTGTTG	161
		R:AGGTTGAAACAGAAGCCGC	
*APOA1*	XM_046932309.1	F:CGCATTCGGGATATGGTGGA	102
		R:GTCAAGCTGTTTGCCCACAG	
*SREBP-1*	AY029224	F: GCAGAAGAGCAAGTCCCTCAA	104
		R: TCGGCATCTCCATCACCTC	

a F, forward primer; R, reverse primer.

b SIRT1, sirtuin1; NRF2, nuclear factor erythroid 2-related factor 2; HO-1, heme-oxygenase 1; SOD1, superoxide dismutase 1; SOD2, superoxide dismutase 2; CAT, catalase; GPX1, glutathione peroxidase 1; CPT-1, carnitine palmitoyl-transferase 1; PPARα, peroxisome proliferator-activated receptor α; APOA1, apolipoprotein A1; SREBP-1, sterol regulatory element-binding protein-1.

### Statistical analysis

The replicate was regarded the experimental unit to evaluate effects on growth performance, while individual broiler was regarded the experimental unit for other data analyses. Statistical analysis of the data was performed using a *t*-test in SAS 9.4 (Institute Inc., Cary, NC, United States). Normality of the data was evaluated by the Shapiro–Wilk statistic (*W* > 0.05). Results are expressed as the mean ± standard error and as plots or graphs. Statistically differences between the two groups were regarded at *p < 0.05, **p < 0.01, and ***p < 0.001, while #*p* < 0.10 was considered indicative of a trend toward significance.

## Results

### Growth performance

**Figure 1:**
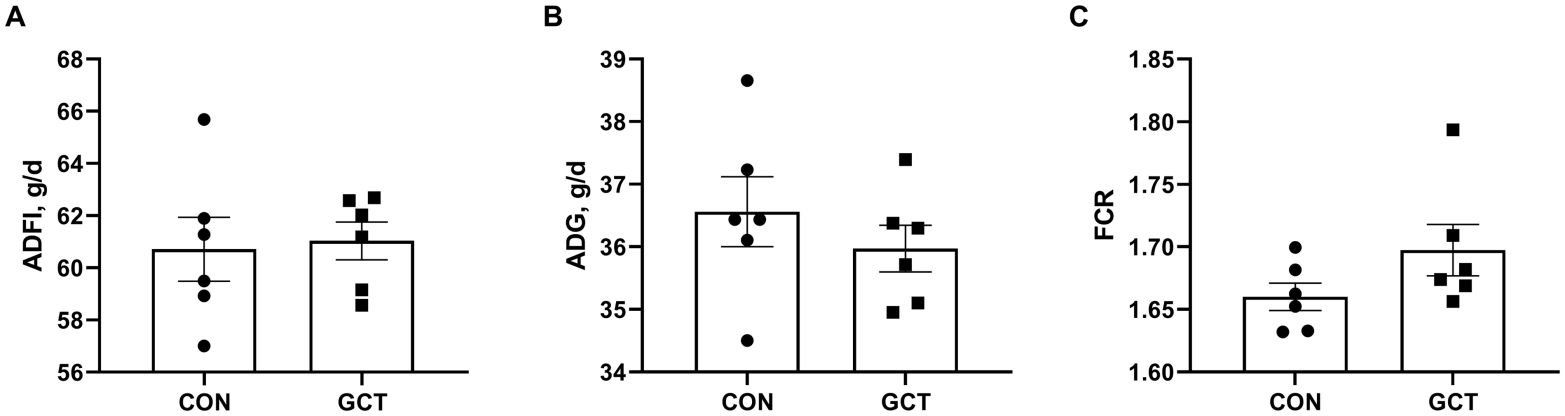
Effect of dietary *Galla Chinensis* tannins (GCT) supplementation on growth performance of broilers. **(A)** average daily feed intake (ADFI); **(B)** average daily gain (ADG); **(C)** feed conversion ratio (FCR). CON, broilers receive a basal diet; GCT, broilers receive a basal diet supplemented with 300 mg/kg microencapsulated GCT. Values are presented as mean ± standard error (*n* = 6).

Effects of dietary microencapsulated GCT addition on the growth performance of broilers are displayed in Figure 1. Throughout the 21 days trial, no significant differences in ADFI (Figure 1A), ADG (Figure 1B), or FCR (Figure 1C) between broilers in the CON and GCT groups (*p* > 0.05).

### Serum biochemical parameters

**Figure 2:**
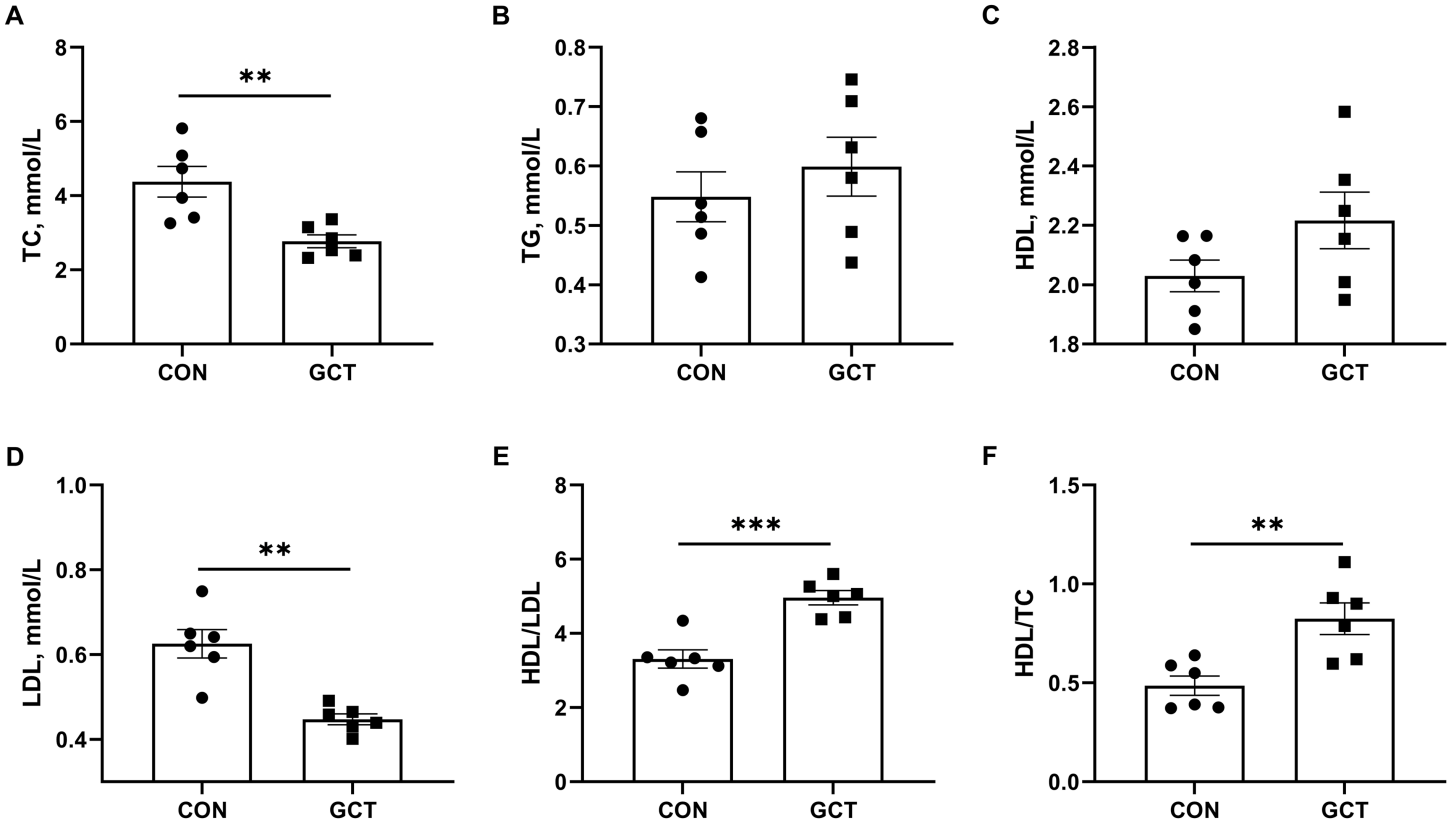
Effects of dietary *Galla Chinensis* tannins (GCT) supplementation on serum biochemical parameters of broilers. **(A)** total cholesterol (TC); **(B)** triglyceride (TG); **(C)** high-density lipoprotein (HDL); **(D)** low-density lipoprotein (LDL); **(E)** HDL/LDL ratio; **(F)** HDL/TC ratio. CON, broilers receive a basal diet; GCT, broilers receive a basal diet supplemented with 300 mg/kg microencapsulated GCT. Values are presented as mean ± standard error (*n* = 6). **p < 0.01 and ***p < 0.001.

As shown in Figure 2, the serum concentrations of TC (Figure 2A) and LDL (Figure 2D) in the GCT group were significantly lower than those in the CON group (*p* < 0.05). Additionally, the GCT group exhibited significantly higher HDL/LDL ratio (Figure 2E) and HDL/TC ratio (Figure 2F) compared with the CON group (*p* < 0.05). No significant differences were observed in serum TG (Figure 2C) and HDL (Figure 2D) concentrations between the two groups (*p* > 0.05).

### Serum and liver antioxidant capacity

**Figure 3:**
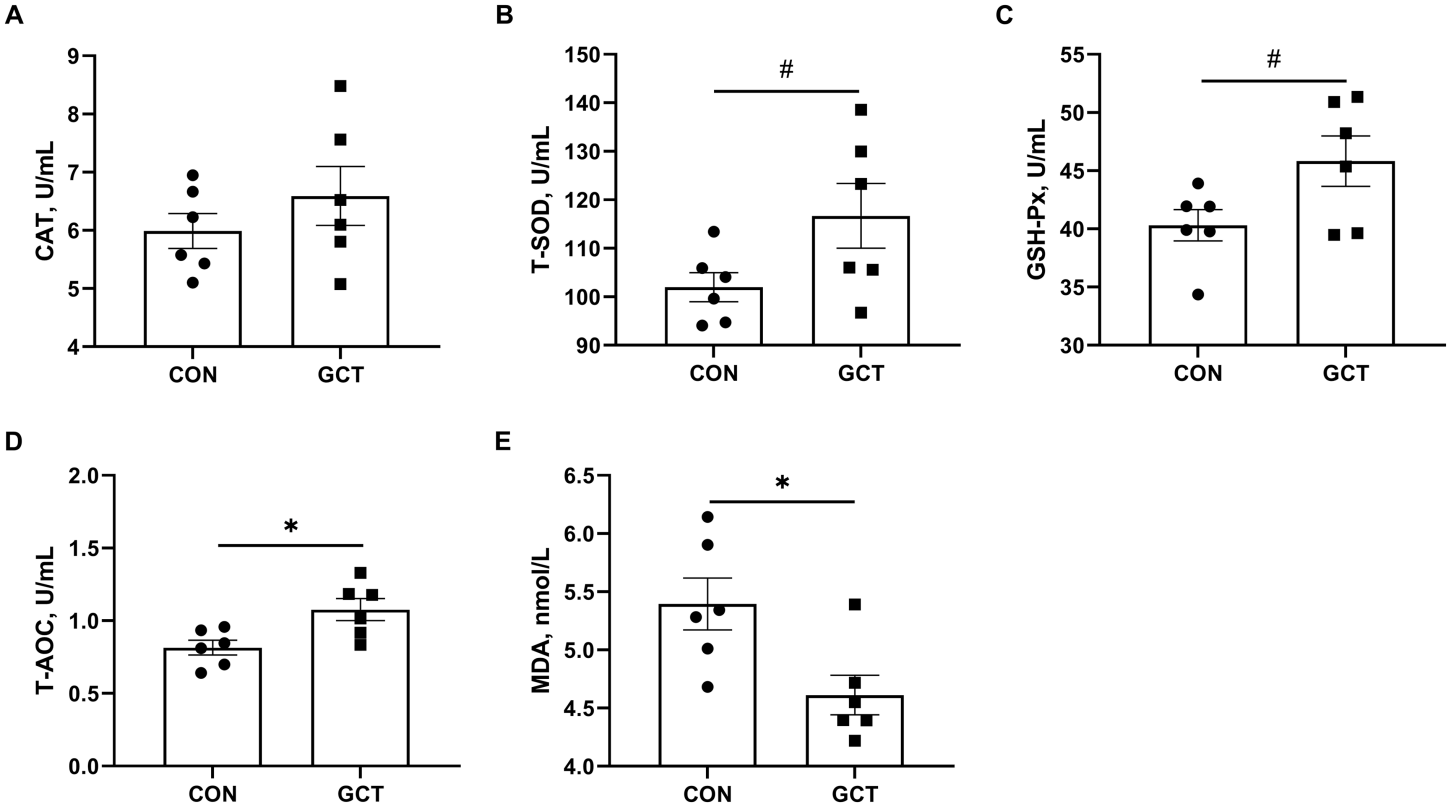
Effects of dietary *Galla Chinensis* tannins (GCT) supplementation on serum antioxidant capacity of broilers. **(A)** catalase (CAT); **(B)** total superoxide dismutase (T-SOD); **(C)** glutathione peroxidase (GSH-Px); **(D)** total antioxidative capacity (T-AOC); **(E)** malondialdehyde (MDA). CON, broilers receive a basal diet; GCT, broilers receive a basal diet supplemented with 300 mg/kg microencapsulated GCT. Values are presented as mean ± standard error (*n* = 6). #*p* < 0.10, *p < 0.05, and **p < 0.01.

Effects of GCT addition on serum antioxidant parameters of broilers are presented in Figure 3. Broilers in the GCT group showed a significantly higher (*p* < 0.05) T-AOC (Figure 3D) level and a lower (*p* < 0.05) MDA (Figure 3E) level. Moreover, there was a trend towards higher serum T-SOD (Figure 3B) and GSH-Px (Figure 3C) activities in the GCT group compared to the CON group (*p* < 0.10). There was no significant difference in serum CAT (Figure 3A) activity (*p* > 0.05).

**Figure 4:**
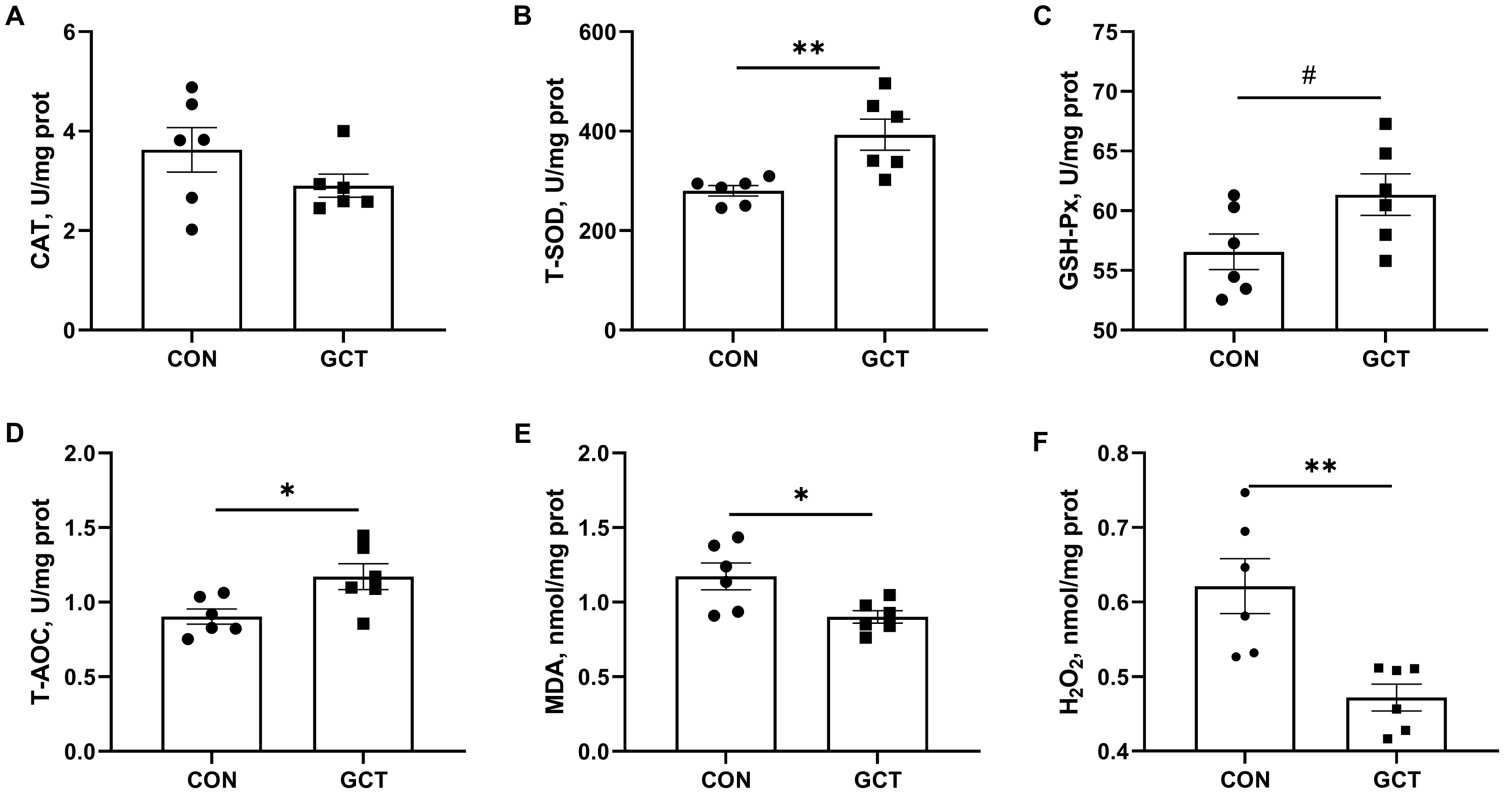
Effects of dietary *Galla Chinensis* tannins (GCT) supplementation on liver antioxidant capacity of broilers. **(A)** catalase (CAT); **(B)** total superoxide dismutase (T-SOD); **(C)** glutathione peroxidase (GSH-Px); **(D)** total antioxidative capacity (T-AOC); **(E)** malondialdehyde (MDA); **(F)** hydrogen peroxide (H_2_O_2_). CON, broilers receive a basal diet; GCT, broilers receive a basal diet supplemented with 300 mg/kg microencapsulated GCT. Values are presented as mean ± standard error (*n* = 6). #*p* < 0.10, *p < 0.05, and **p < 0.01.

Effects of GCT addition on hepatic antioxidant parameters of broilers are shown in Figure 4. Dietary 300 mg/kg microencapsulated GCT addition significantly elevated (*p* < 0.05) T-SOD (Figure 4B) and T-AOC (Figure 4D) levels, while significantly decreased (*p* < 0.05) MDA (Figure 4E) and H_2_O_2_ (Figure 4F) concentrations in the liver of broilers. Moreover, microencapsulated GCT supplementation showed a tendency toward increased (*p* < 0.10) hepatic GSH-Px activity (Figure 4C). There was no significant difference observed in liver CAT (Figure 4A) activity between the two groups (*p* > 0.05).

### Liver lipid metabolism

**Figure 5:**
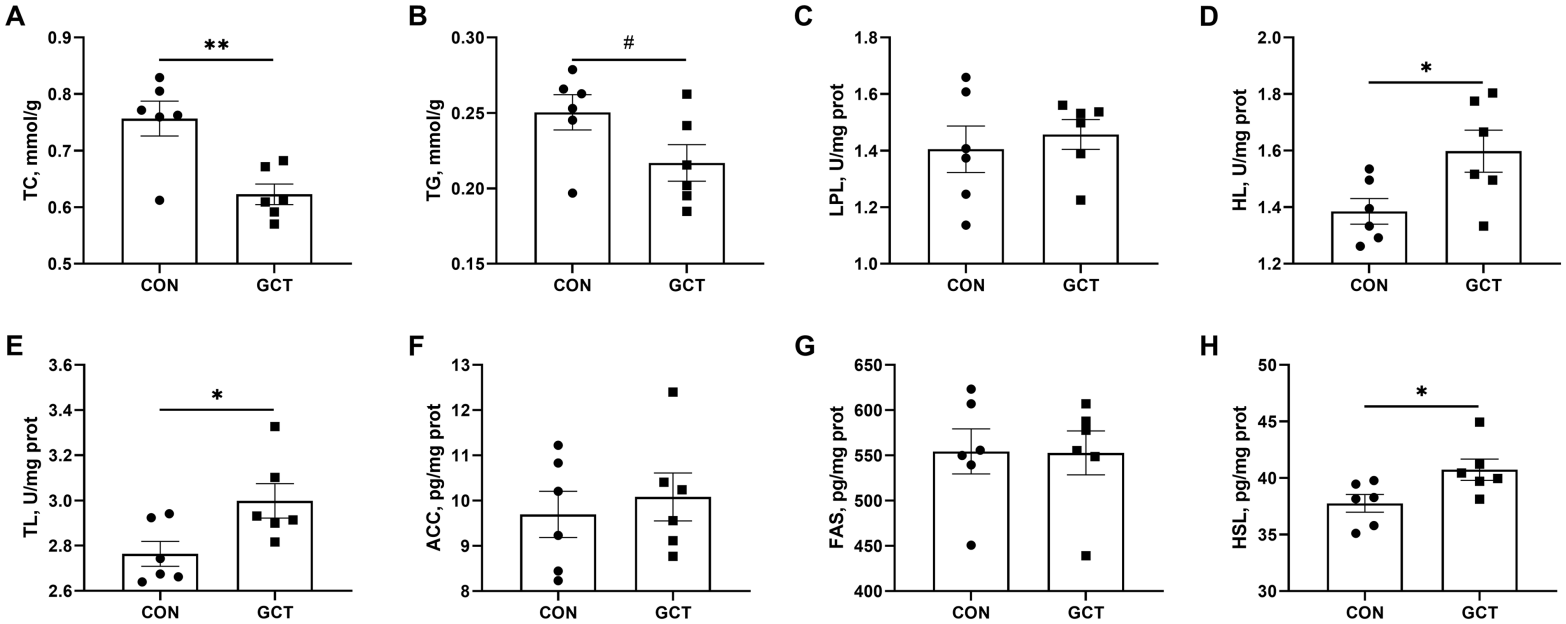
Effects of dietary *Galla Chinensis* tannins (GCT) supplementation on liver lipid metabolism of broilers. **(A)** total cholesterol (TC); **(B)** triglyceride (TG); **(C)** lipoprotein lipase (LPL); **(D)** hepatic lipase (HL); **(E)** total lipase (TL); **(F)** acetyl CoA carboxylase (ACC); **(G)** fatty acid synthase (FAS); **(H)** hormone-sensitive triglyceride lipase (HSL). CON, broilers receive a basal diet; GCT, broilers receive a basal diet supplemented with 300 mg/kg microencapsulated GCT. Values are presented as mean ± standard error (*n* = 6). #*p* < 0.10, *p < 0.05, and **p < 0.01.

In the liver, the GCT group exhibited significantly lower (*p* < 0.05) TC (Figure 5A) level and significantly higher (*p* < 0.05) activities of HL (Figure 5D), TL (Figure 5E), and HSL (Figure 5H) relative to the CON group. Furthermore, GCT supplementation to broiler diet showed a trend (*p* < 0.10) toward reduced liver TG (Figure 5B) level compared with CON broilers. Dietary GCT supplementation had no remarkable influences (*p* > 0.05) on the activities of LPL (Figure 5C), ACC (Figure 5F), and FAS (Figure 5G) in the liver.

### Liver histolomorph

**Figure 6:**
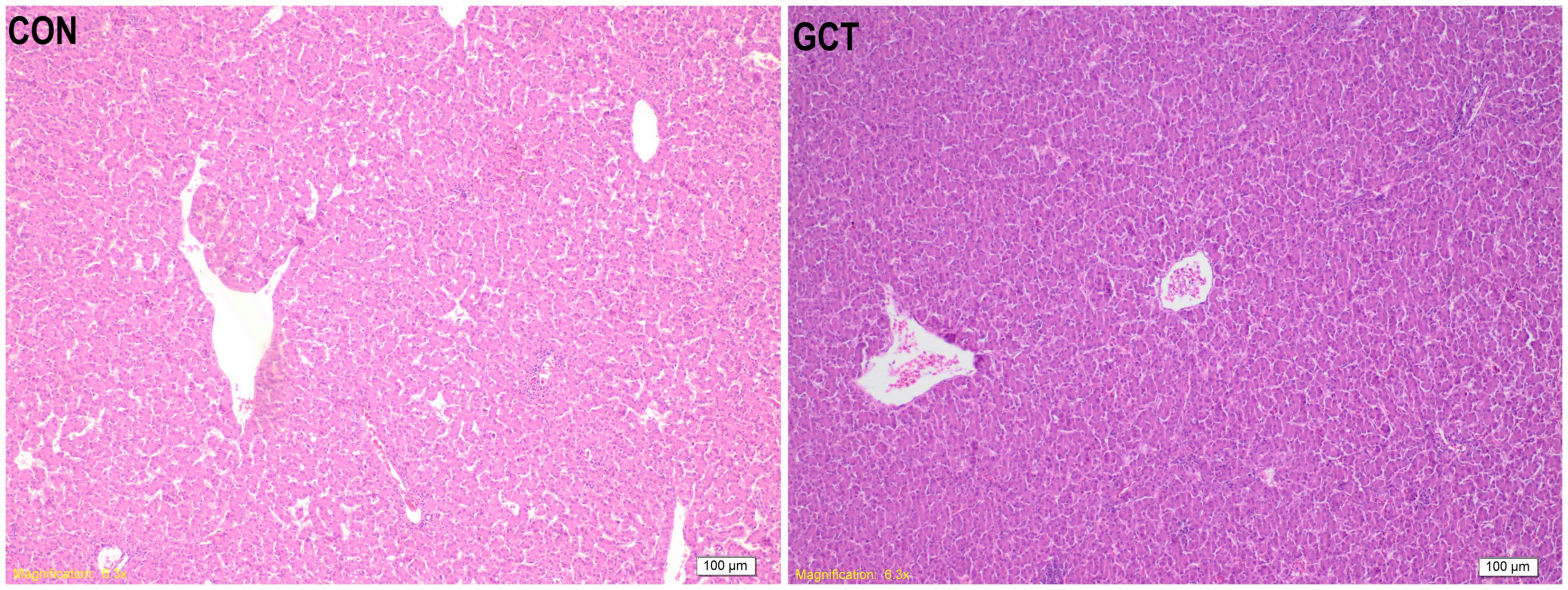
Hematoxylin and eosin photomicrographs of liver tissues. CON, broilers receive a basal diet; GCT, broilers receive a basal diet supplemented with 300 mg/kg microencapsulated GCT.

As shown in Figure 6, the liver structure of the CON group and GCT group appeared intact, characterized by well-organized liver cell cords and normal morphology of liver cells. The bile duct structure in the portal area was clear, without any observed changes such as bile duct proliferation, lumen enlargement, or inflammatory cell infiltration. However, in the GCT group, the liver sinus appeared thinner and more compact, accompanied by a noticeable reduction in liver tissue fat globules and a tendency towards decreased sphericity.

### Expressions of hepatic antioxidant-related genes

**Figure 7:**
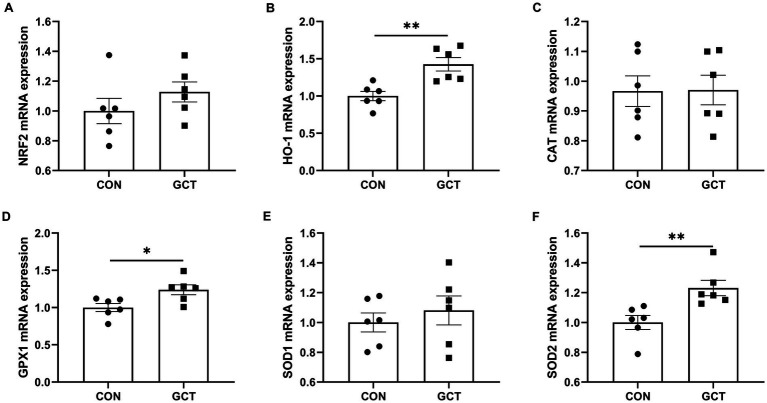
Effects of dietary *Galla Chinensis* tannins (GCT) supplementation on mRNA expressions of hepatic antioxidant-related genes of broilers. **(A)** nuclear factor erythroid 2-related factor 2 (NRF2); **(B)** heme-oxygenase 1 (HO-1); **(C)** catalase (CAT); **(D)** glutathione peroxidase 1 (GPX1); **(E)** superoxide dismutase 1 (SOD1); **(F)** superoxide dismutase 2 (SOD2). CON, broilers receive a basal diet; GCT, broilers receive a basal diet supplemented with 300 mg/kg microencapsulated GCT. Values are presented as mean ± standard error (*n* = 6). *p < 0.05 and **p < 0.01.

Effects of GCT addition on relative expressions of hepatic antioxidant-related genes of broilers are shown in Figure 7. The GCT group exhibited significantly upregulated (*p* < 0.05) mRNA expressions of *HO-1* (Figure 7B), *GPX1* (Figure 7D), and *SOD2* (Figure 7F) compared in the CON group (Figure 6). However, dietary GCT supplementation had no significantly affects (*p* > 0.05) on the mRNA expressions of *NRF2* (Figure 7A), *CAT* (Figure 7C), and *SOD1* (Figure 7E).

### Expressions of hepatic lipid metabolism-related genes

**Figure 8:**
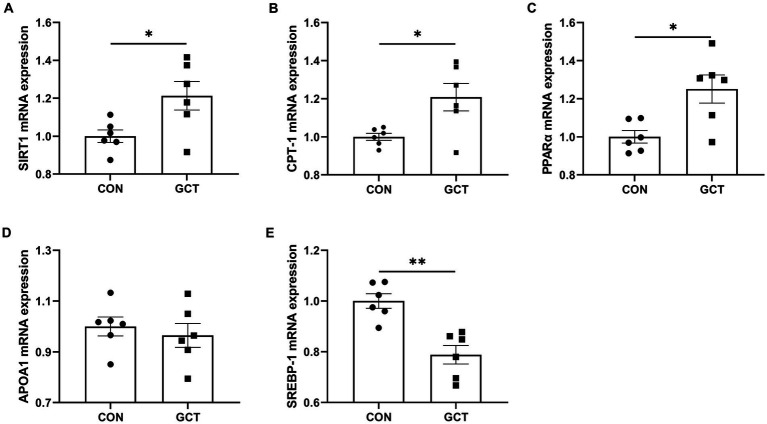
Effects of dietary *Galla Chinensis* tannins (GCT) supplementation on mRNA expressions of hepatic lipid metabolism-related genes of broilers. **(A)** Sirtuin1 (SIRT1); **(B)** carnitine palmitoyl-transferase 1 (CPT-1); **(C)** peroxisome proliferator-activated receptor α (PPARα); **(D)** apolipoprotein A1 (APOA1); **(E)** sterol regulatory element-binding protein-1 (SREBP-1). CON, broilers receive a basal diet; GCT, broilers receive a basal diet supplemented with 300 mg/kg microencapsulated GCT. Values are presented as mean ± standard error (*n* = 6). *p < 0.05 and **p < 0.01.

As shown in Figure 8, dietary microencapsulated GCT supplementation significantly upregulated (*p* < 0.05) the hepatic mRNA expressions of *SIRT1* (Figure 8A), *CPT-1* (Figure 8B), and *PPARα* (Figure 8C), while significantly downregulated (*p* < 0.05) the mRNA expression of *SREBP-1* (Figure 8E) in broilers. No significant difference was found (*p* > 0.05) in hepatic *APOA1* (Figure 8D) mRNA expression between CON and GCT groups.

## Discussion

In current study, dietary supplementation with 300 mg/kg microencapsulated GCT did not show negative effect on the growth performance of young broilers, which was in accordance with previous studies (18, 24). Tannins in higher concentration are antinutritional because made chelates and reduce protein digestibility (25). It was reported that tannins could form complexes with proteins (both enzymes and nonenzyme proteins) to form tannin-protein complexes, reducing the bioavailability of nutrients in the feed (26). Buyse et al. (24) reported that broilers fed a diet containing higher dose (2000 mg/kg) of chestnut tannins had lower performance during the grower and finisher phases than broilers fed a diet with a lower dose (500 mg/kg) of chestnut tannins. These results indicated that low concentrations of tannins had no adverse effects on broiler growth performance. Additionally, tannins have poor palatability for livestock. Microencapsulation is widely used in the feed industry to modify the absorption site and mask undesirable taste and odor (17). In piglets, dietary supplementation of microencapsulated tannins extracted from GC had no adverse effects on growth performance (27). Furthermore, Niu et al. (18) indicated that 300 mg/kg microencapsulated CGT improved the feed conversion ratio of broilers throughout the trial, which could be attributed to the improved intestinal development after 42 days of feeding (28). Therefore, supplementing 300 mg/kg microencapsulated CGT in the diet had no adverse effects on the growth performance of young broiler chickens.

The liver, a vital metabolic organ, plays an important role in metabolism and defense against bacterial invasion and bacterial products (22). It contains a large number of mitochondria and acts as a regulator of energy balance with high oxygen consumption and reactive oxygen species production (29). Intensive farming practices in poultry production have increased the likelihood of hepatic oxidative injury because of higher metabolic demands during the growth and development of broilers (30). Therefore, the implementation of measures is necessary to improve the antioxidant capacity of the liver. In the current study, supplementation of 300 mg/kg GCT elevated T-SOD and GSH-Px activities, as well as T-AOC levels, and decreased MDA levels in the serum and liver. Additionally, it led to a reduction in H_2_O_2_ concentration specially within the liver. Antioxidant enzymes, such as SOD, GSH-Px, and CAT, play vital roles in preventing oxidative damage (22). The SOD converts ROS to the H_2_O_2_, which can then be degraded into water and oxygen by GSH-Px and CAT (31). T-AOC is an important integrative index reflecting the total antioxidant status (32), while MDA is an important indicator of detecting the degree of lipid peroxidation of the body (33, 34). Higher concentrations of H_2_O_2_ and MDA are typically associated with cellular damage. A previous study indicated that tannic acid improved CAT, SOD, and GSH-Px activities in rats challenged by arsenic trioxide (35). Song et al. (36) found that dietary supplementation of GCT improved intestinal antioxidant capacity in weaned piglets. Furthermore, GCT supplementation upregulated the mRNA expression of *HO-1* in the liver of broilers in the current study. HO-1, which is induced as a crucial stress protein, regulates many antioxidant enzymes and proteasomes and plays a major role in regulating intracellular ROS levels to mitigate cellular oxidative stress response due to its antioxidant effects (37, 38). Consistently, hepatic GPX1 and SOD2 expressions was upregulated in CGT broilers. GPX1 and SOD2 are genes that encode the antioxidant enzymes GSH-Px and SOD, respectively. Above all, our findings demonstrated that dietary supplementation of 300 mg/kg microencapsulated GCT enhanced the antioxidant capacity of young broilers through upregulating the HO-1 expression and improving the activities of antioxidant enzymes.

Antioxidant capacity of the body is generally associated with lipid metabolism. Our study indicated that dietary GCT addition reduced the concentrations of TC and LDL, and increased the HDL/LDL ratio and HDL/TC ratio in the serum. Other researchers also found that tannins supplementation decreased serum levels of LDL, TC, and TG, but increased serum level of HDL (39, 40). It is well known that low blood HDL level and high TG, TC, and LDL levels are the major risk factors for cerebrovascular and cardiovascular diseases (41). Excessive accumulation of TG and TC is often associated with liver damage (42). The HDL and LDL are apolipoproteins that represent different forms of lipid transport in the blood. Specifically, HDL facilitates the transportation of excess cholesterol from the surrounding tissues back to the liver for eventual excretion from the body, while LDL transports cholesterol from the liver to various tissues of the body (43, 44). Therefore, the serum HDL/LDL ratio and HDL/TC ratios serve as reliable indicators of lipid accumulation. Not surprisingly, lower TC concentration in the liver was found in GCT broilers compared with the CON broilers, which might be also related to the increased TL, HL, and HSL activities in the liver. Studies have demonstrated that HL facilitates the absorption of unesterified cholesterol that has accumulated in HDL, and also plays a role in catalyzing the breakdown of TG into fatty acids (45). The HSL, serving as a key enzyme in fat breakdown, accelerates the hydrolysis of TG to glycerol and fatty acids (46). Kwon et al. (47) indicated that GC extract exhibited potent inhibitory activity against lipid accumulation in the pancreasin *in vitro*. Zou et al. (48) also showed that high molecular weight persimmon tannin administration decreased serum TG and free fatty acids concentrations, increased the excretion of TG and TC, and improved hepatic steatosis in rats fed with a high-fat diet. Above all, our findings suggested that supplementation of 300 mg/kg microencapsulated GCT in the diet benefited to increase lipid synthesis and accelerate lipolysis in young broilers.

Lipid accumulation is a complex process regulated by numerous gene expression alterations that control lipolysis and lipogenesis (49). The SIRT1, CPT-1, and CPT-2 play a major role in fatty acid β-oxidation. Our study showed that dietary GCT addition significantly upregulated hepatic *SIRT1* and *CPT-1* mRNA expression in broiler chickens. SIRT1 governs the regulation of PGC-1α, which subsequently modulates fatty acid oxidation and facilitates fatty acid catabolism in the liver (50, 51). CPT-1 is considered a rate-limiting enzyme involved in transporting fatty acids into the mitochondria for oxidation (52, 53). Besides, GCT enhanced the *PPARα* mRNA expression and weakened *SREBP-1* mRNA expression in our study. PPARα is a ligand-activated nuclear receptor, which mainly influences fatty acid metabolism and modulates the lipid accumulation through increasing LPL expression (54, 55). SREBP-1 is a transcriptional regulator of genes involved in fatty acid and TG syntheses, and it catalyzes the transcription of the key genes including FAS and ACC (56, 57). Similarly, Zou et al. (48) also demonstrated that high molecular weight persimmon tannin decreased expression of FAS, SREBP-1, and ACC, increased the expression of CPT-1, and stimulated AMP-activated protein kinase phosphorylation in the liver of rats fed a high-fat diet. These results revealed that microencapsulated GCT might improve liver lipid accumulation by activating the SIRT1/SREBP-1 pathway and increasing the expression of genes related to fatty acid β-oxidation.

## Conclusion

In conclusion, dietary supplementation with 300 mg/kg microencapsulated GCT enhanced the antioxidant capacity and improved the lipid metabolism of young broilers although had no effects on the growth performance. These findings provide valuable references for the utilization of GCT to alleviate oxidative stress and lipid metabolic disorders, and support the utilization of tannins extracted from GC in the poultry industry.

## Data availability statement

The original contributions presented in the study are included in the article/supplementary material, further inquiries can be directed to the corresponding authors.

## Ethics statement

The animal studies were approved by the Animal Care and Use Committee of Shandong Agricultural University (protocol code SDAUA-2022-073). The studies were conducted in accordance with the local legislation and institutional requirements. Written informed consent was obtained from the owners for the participation of their animals in this study.

## Author contributions

XR: Conceptualization, Data curation, Formal analysis, Methodology, Software, Visualization, Writing – original draft. PY: Conceptualization, Data curation, Formal analysis, Software, Visualization, Writing – review & editing. JN: Data curation, Investigation, Methodology, Project administration, Writing – review & editing. YLiu: Data curation, Investigation, Methodology, Project administration, Writing – review & editing. YLi: Conceptualization, Methodology, Resources, Validation, Writing – review & editing. LH: Methodology, Resources, Writing – review & editing. SJ: Investigation, Validation, Writing – review & editing. NJ: Formal analysis, Writing – review & editing. XY: Visualization, Writing – review & editing. JL: Funding acquisition, Investigation, Resources, Supervision, Writing – review & editing. WY: Conceptualization, Funding acquisition, Methodology, Resources, Supervision, Validation, Writing – review & editing.

## References

[1] Kpomasse CC Oke OE Houndonougbo FM Tona K. Broiler production challenges in the tropics: a review. Vet Med Sci. (2021) 7:831–42. doi: 10.1002/vms3.435, PMID: 33559980 PMC8136938

[2] Farahat M Abdallah F Abdel-Hamid T Hernandez-Santana A. Effect of supplementing broiler chicken diets with green tea extract on the growth performance, lipid profile, antioxidant status and immune response. B. Poult Sci. (2016) 57:714–22. doi: 10.1080/00071668.2016.1196339, PMID: 27302855

[3] Han HL Zhang JF Yan EF Shen MM Wu JM Gan ZD. Effects of taurine on growth performance, antioxidant capacity and lipid metabolism in broiler chickens. Poult Sci. (2020) 99:5707–17. doi: 10.1016/j.psj.2020.07.020 33142488 PMC7647726

[4] Panda AK Cherian G. Role of vitamin E in counteracting oxidative stress in poultry. J Poult Sci. (2014) 51:109–17. doi: 10.2141/jpsa.0130134

[5] Meng J Ma N Liu H Liu J Liu J Wang J. Untargeted and targeted metabolomics profifiling reveals the underlying pathogenesis and abnormal arachidonic acid metabolism in laying hens with fatty liver hemorrhagic syndrome. Poult Sci. (2021) 100:101320. doi: 10.1016/j.psj.2021.101320, PMID: 34274572 PMC8319003

[6] Abdulla NR Loh TC Foo HL Alshelmani MI Akit H. Influence of dietary ratios of n-6: n-3 fatty acid on gene expression, fatty acid profile in liver and breast muscle tissues, serum lipid profile, and immunoglobulin in broiler chickens. J Appl Poult Res. (2019) 28:454–69. doi: 10.3382/japr/pfz008

[7] Chen D Ran D Wang C Liu Y Ma Y Song R. Role of mitochondrial dysfunction and PINK1/Parkin-mediated mitophagy in cd-induced hepatic lipid accumulation in chicken embryos. Life Sci. (2021) 284:119906. doi: 10.1016/j.lfs.2021.119906, PMID: 34478761

[8] Mutinati M Pantaleo M Roncetti M Piccinn M Rizzo A Sciorsci R. Oxidative stress in neonatology. A review. Reprod Domest Anim. (2014) 49:7–16. doi: 10.1111/rda.12230 24112309

[9] Kairalla MA Alshelmani MI Aburas AA. Effect of diet supplemented with graded levels of garlic (Allium sativum L.) powder on growth performance, carcass characteristics, blood hematology, and biochemistry of broilers. Open Vet J. (2022) 12:595–601. doi: 10.5455/OVJ.2022.v12.i5.1, PMID: 36589396 PMC9789753

[10] Kairalla MA Aburas AA Alshelmani MI. Effect of diet supplemented with graded levels of ginger (Zingiber officinale) powder on growth performance, hematological parameters, and serum lipids of broiler chickens. Arch Razi Inst. (2022) 77:2089–95. doi: 10.22092/ARI.2022.359958.2524, PMID: 37274916 PMC10237567

[11] Abouelmagd SA Abd Ellah NH Amen O Abdelmoez A Mohamed NG. Self-assembled tannic acid complexes for pH-responsive delivery of antibiotics: role of drug-carrier interactions. Int J Pharm. (2019) 562:76–85. doi: 10.1016/j.ijpharm.2019.03.009, PMID: 30851388

[12] Besharati M Maggiolino A Palangi V Kaya A Jabbar M Eseceli H. Tannin in ruminant nutrition: review. Molecules. (2022) 27:8273. doi: 10.3390/molecules27238273, PMID: 36500366 PMC9738529

[13] Ren Y Zhang X Li T Zeng Y Wang J Huang Q. Galla Chinensis, a traditional Chinese medicine: comprehensive review of botany, traditional uses, chemical composition, pharmacology and toxicology. J Ethnopharmacol. (2021) 278:114247. doi: 10.1016/j.jep.2021.114247, PMID: 34052353

[14] Huang Q Liu X ZhaoG T Wang Y. Potential and challenges of tannins as an alternative to in-feed antibiotics for farm animal production. Anim Nutr. (2018) 4:137–50. doi: 10.1016/j.aninu.2017.09.004, PMID: 30140753 PMC6104569

[15] Li Y Zhu L Guo C Xue M Xia F Wang Y. Dietary intake of hydrolyzable tannins and condensed tannins to regulate lipid metabolism. Mini Rev Med Chem. (2022) 22:1789–802. doi: 10.2174/1389557522666211229112223, PMID: 34967286

[16] Caprarulo V Hejna M Giromini C Liu Y Dell’Anno M Sotira S. Evaluation of dietary administration of chestnut and quebracho tannins on growth, serum metabolites and fecal parameters of weaned piglets. Animals. (2020) 10:1945. doi: 10.3390/ani10111945, PMID: 33105748 PMC7690424

[17] Wang M Huang H Hu Y Huang J Yang H Wang L. Effects of dietary microencapsulated tannic acid supplementation on the growth performance, intestinal morphology, and intestinal microbiota in weaning piglets. J Anim Sci. (2020) 98:skaa112. doi: 10.1093/jas/skaa112, PMID: 32255185 PMC7199885

[18] Niu J Wang Q Jing C Liu Y Liu H Jiao N. Dietary Galla Chinensis tannic acid supplementation in the diets improves growth performance, immune function and liver health status of broiler chicken. Front Vet Sci. (2022) 9:1024430. doi: 10.3389/fvets.2022.1024430, PMID: 36311675 PMC9614106

[19] Yuan P Xu H Ma H Niu J Liu Y Huan L. Effects of dietary Galla Chinensis tannin supplementation on immune function and liver health in broiler chickens challenged with lipopolysaccharide. Front Vet Sci. (2023) 10:1126911. doi: 10.3389/fvets.2023.1126911, PMID: 36865438 PMC9974168

[20] National Research Council. Nutrient requirements of poultry. Washington: 9th National Academy Press (1994).

[21] Chen J Li F Yang W Jiang S Li Y. Supplementation with exogenous catalase from penicillium notatum in the diet ameliorates lipopolysaccharide-induced intestinal oxidative damage through affecting intestinal antioxidant capacity and microbiota in weaned pigs. Microbiol Spectr. (2021) 9:e0065421. doi: 10.1128/Spectrum.00654-21, PMID: 34908474 PMC8672903

[22] Li Y Zhao X Jiang X Chen L Hong L Zhuo Y. Effects of dietary supplementation with exogenous catalase on growth performance, oxidative stress, and hepatic apoptosis in weaned piglets challenged with lipopolysaccharide. J Anim Sci. (2020) 98:67. doi: 10.1093/jas/skaa067, PMID: 32152634 PMC7205395

[23] Zhang P Jing C Liang M Jiang S Huang L Jiao N. Zearalenone exposure triggered cecal physical barrier injury through the TGF-β1/Smads signaling pathway in weaned piglets. Toxins. (2021) 13:902. doi: 10.3390/toxins13120902, PMID: 34941739 PMC8708673

[24] Buyse K Delezie E Goethals L Van Noten N Ducatelle R Janssens GPJ. Chestnut tannins in broiler diets: performance, nutrient digestibility, and meat quality. Poult Sci. (2021) 100:101479. doi: 10.1016/j.psj.2021.101479, PMID: 34700100 PMC8554258

[25] Alshelmani MI Abdalla EA Kaka U Basit MA. Nontraditional feedstuffs as an alternative in poultry feed, Advances in poultry nutrition research. IntechOpen, (2021).

[26] Maugeri A Lombardo GE Cirmi S Süntar I Barreca D Laganà G. Pharmacology and toxicology of tannins. Arch Toxicol. (2022) 96:1257–77. doi: 10.1007/s00204-022003250-0 35199243

[27] Wang WM Huang HJ Liu S Zhuang Y Yang HS Li LY. Tannic acid modulates intestinal barrier functions associated with intestinal morphology, antioxidative activity, and intestinal tight junction in a diquat-induced mouse model. RSC Adv. (2019) 9:31988–98. doi: 10.1039/c9ra04943f, PMID: 35530805 PMC9072718

[28] Jing C Niu J Liu Y Jiao N Huang L Jiang S. Tannic acid extracted from Galla Chinensis supplementation in the diet improves intestinal development through suppressing inflammatory responses via blockage of NF-κB in broiler chickens. Animals. (2022) 12:2397. doi: 10.3390/ani12182397, PMID: 36139256 PMC9495145

[29] Mansouri M Berger P. Multigene delivery in mammalian cells: recent advances and applications. Biotechnol Adv. (2018) 36:871–9. doi: 10.1016/j.biotechadv.2018.01.012 29374595

[30] Liu Y Li Y Niu J Liu H Jiao N Huang L. Effects of dietary Macleaya cordata extract containing isoquinoline alkaloids supplementation as an alternative to antibiotics in the diets on growth performance and liver health of broiler chickens. Front Vet Sci. (2022) 9:950174. doi: 10.3389/fvets.2022.950174, PMID: 35968000 PMC9363708

[31] Ali SS Ahsan H Zia MK Siddiqui T Khan FH. Understanding oxidants and antioxidants: classical team with new players. J Food Biochem. (2020) 44:e13145. doi: 10.1111/jfbc.13145, PMID: 31960481

[32] Li Y Liu H Zhang L Yang Y Lin Y Zhuo Y. Maternal dietary fiber composition during gestation induces changes in offspring antioxidative capacity, inflammatory response, and gut microbiota in a sow model. Int J Mol Sci. (2020) 21:31. doi: 10.3390/ijms21010031, PMID: 31861629 PMC6981455

[33] Mishra B Jha R. Oxidative stress in the poultry gut: potential challenges and interventions. Front Vet Sci. (2019) 6:60. doi: 10.3389/fvets.2019.00060, PMID: 30886854 PMC6409315

[34] Li H Song F Duan LR Sheng JJ Xie YH Yang Q. Paeonol and danshensu combination attenuates apoptosis in myocardial infarcted rats by inhibiting oxidative stress, roles of Nrf2/HO-1 and PI3K/Akt pathway. Sci Rep. (2016) 6:23693. doi: 10.1038/srep23693, PMID: 27021411 PMC4810373

[35] Jin W Xue Y Xue Y Han X Song Q Zhang J. Tannic acid ameliorates arsenic trioxide-induced nephrotoxicity, contribution of NF-κB and Nrf2 pathways. Biomed Pharmacother. (2020) 126:110047. doi: 10.1016/j.biopha.2020.110047, PMID: 32146384

[36] Song Y Luo Y Yu B He J Zheng P Mao X. Tannic acid extracted from gallnut prevents post-weaning diarrhea and improves intestinal health of weaned piglets. Anim Nutr. (2021) 7:1078–86. doi: 10.1016/j.aninu.2021.04.005, PMID: 34738038 PMC8546364

[37] Bao L Li J Zha D Zhang L Gao P Yao T. Chlorogenic acid prevents diabetic nephropathy by inhibiting oxidative stress and inflammation through modulation of the Nrf2/HO-1 and NF-kB pathways. Int Immunopharmacol. (2018) 54:245–53. doi: 10.1016/j.intimp.2017.11.021 29161661

[38] Loboda A Damulewicz M Pyza E Jozkowicz A Dulak J. Role of Nrf2/HO-1 system in development, oxidative stress response and diseases: an evolutionarily conserved mechanism. Cell Mol Life Sci. (2016) 73:3221–47. doi: 10.1007/s00018-016-2223-0, PMID: 27100828 PMC4967105

[39] Manzoor F Nisa MU Hussain HA Ahmad N Umbreen H. Effect of different levels of hydrolysable tannin intake on the reproductive hormones and serum biochemical indices in healthy female rats. Sci Rep. (2020) 10:20600. doi: 10.1038/s41598-020-77672-0, PMID: 33244120 PMC7692459

[40] Sieniawska E. Activities of tannins-from in vitro studies to clinical trials. Nat Prod Commun. (2015) 10:1877–84. doi: 10.1177/1934578X1501001118, PMID: 26749816

[41] Duran EK Aday AW Cook NR Buring JE Ridker PM Pradhan AD. Triglyceride-rich lipoprotein cholesterol, small dense LDL cholesterol, and incident cardiovascular disease. J Am Coll Cardiol. (2020) 75:2122–35. doi: 10.1016/j.jacc.2020.02.059, PMID: 32354380 PMC8064770

[42] Xie C Chen Z Zhang C Xu X Jin J Zhan W. Dihydromyricetin ameliorates oleic acidinduced lipid accumulation in L02 and HepG2 cells by inhibiting lipogenesis and oxidative stress. Life Sci. (2016) 157:131–9. doi: 10.1016/j.lfs.2016.06.001, PMID: 27265384

[43] Scott Kiss R Sniderman A. Shunts, channels and lipoprotein endosomal traffic: a new model of cholesterol homeostasis in the hepatocyte. J Biomed Res. (2017) 31:95–107. doi: 10.7555/JBR.31.20160139, PMID: 28808191 PMC5445212

[44] Yin DF Tong TJ Moss Amy F Zhang RY Kuang YG Zhang Y. Effects of coated trace minerals and the fat source on growth performance, antioxidant status, and meat quality in broiler chickens. J Poult Sci. (2022) 59:56–63. doi: 10.2141/jpsa.0200108, PMID: 35125913 PMC8791779

[45] Gu T Duan M Liu J Chen L Tian Y Xu W. Effects of tributyrin supplementation on liver fat deposition, lipid levels and lipid metabolism-related gene expression in broiler chickens. Genes. (2022) 13:2219. doi: 10.3390/genes13122219, PMID: 36553486 PMC9777756

[46] Recazens E Mouisel E Langin D. Hormone-sensitive lipase: sixty years later. Prog Lipid Res. (2021) 82:101084. doi: 10.1016/j.plipres.2020.101084, PMID: 33387571

[47] Kwon OJ Bae S Lee HY Hwang JY Lee EW Ito H. Pancreatic lipase inhibitory gallotannins from Galla rhois with inhibitory effects on adipocyte differentiation in 3T3-L1 cells. Molecules. (2013) 18:10629–38. doi: 10.3390/molecules180910629, PMID: 24002138 PMC6269876

[48] Zou B Ge Z Zhang Y Du J Xu Z Li CM. Persimmon tannin accounts for hypolipidemic effects of persimmon through activating of AMPK and suppressing NF-κB activation and inflammatory responses in high-fat diet rats. Food Funct. (2014) 5:1536–46. doi: 10.1039/C3FO60635J, PMID: 24841999

[49] Han J Li L Wang D Ma H. (−)-Hydroxycitric acid reduced fat deposition via regulating lipid metabolism-related gene expression in broiler chickens. Lipids Health Dis. (2016) 15:37–50. doi: 10.1186/s12944-016-0208-5, PMID: 26912252 PMC4765117

[50] Xu X Chen X Huang Z Chen D Yu B Chen H. Dietary apple polyphenols supplementation enhances antioxidant capacity andimproves lipid metabolism in weaned piglets. J Anim Physiol Anim Nutr. (2019) 103:1512–20. doi: 10.1111/jpn.13152, PMID: 31268198

[51] Schug TT Li X. Sirtuin 1 in lipid metabolism and obesity. Ann Med. (2011) 43:198–211. doi: 10.3109/07853890.2010.547211, PMID: 21345154 PMC3173813

[52] Liu Y Zhou J Musa BB Khawar H Yang X Cao Y. Developmental changes in hepatic lipid metabolism of chicks during the embryonic periods and the first week of posthatch. Poultry Sci. (2020) 99:1655–62. doi: 10.1016/j.psj.2019.11.004, PMID: 32111330 PMC7587903

[53] Lin M Lv D Zheng Y Wu M Xu C Zhang Q. Downregulation of CPT2 promotes tumorigenesis and chemoresistance to cisplatin in hepatocellular carcinoma. Onco Targets Ther. (2018) 11:3101–10. doi: 10.2147/OTT.S163266, PMID: 29872321 PMC5975610

[54] Sinha RA Rajak S Singh BK Yen PM. Hepatic lipid catabolism via PPARα-lysosomal crosstalk. Int J Mol Sci. (2020) 21:2391. doi: 10.2337/diabetes.51.4.901, PMID: 32244266 PMC7170715

[55] Lian CY Wei S Li ZF Zhuang SH Wang ZY Wang L. Glyphosate-induced autophagy inhibition results in hepatic steatosis via mediating epigenetic reprogramming of PPARα in roosters. Environ Pollut. (2023) 324:121394. doi: 10.1016/j.envpol.2023.121394, PMID: 36906059

[56] Zhu L Du W Liu Y Cheng M Wang X Zhang C. Prolonged high-glucose exposure decreased SREBP-1/FASN/ACC in Schwann cells of diabetic mice via blocking PI3K/Akt pathway. J Cell Biochem. (2019) 120:5777–89. doi: 10.1002/jcb.27864, PMID: 30362584

[57] Wan X Yang Z Ji H Li N Yang Z Xu L. Effects of lycopene on abdominal fat deposition, serum lipids levels and hepatic lipid metabolism-related enzymes in broiler chickens. Anim Biosci. (2021) 34:385–92. doi: 10.5713/ajas.20.0432, PMID: 33152222 PMC7961199

